# Traditional Cardiovascular Risk Factors and Coronary Collateral Circulation: A Meta-Analysis

**DOI:** 10.3389/fcvm.2021.743234

**Published:** 2021-11-03

**Authors:** Junyu Pei, Xiaopu Wang, Zhenhua Xing

**Affiliations:** ^1^Department of Cardiovascular Medicine, The Second Xiangya Hospital, Central South University, Changsha, China; ^2^Department of Emergency Medicine, Second Xiangya Hospital, Central South University, Changsha, China; ^3^Emergency Medicine and Difficult Diseases Institute, Central South University, Changsha, China

**Keywords:** coronary collateral circulation, smoking habit, hypertension, diabetes, meta-analysis

## Abstract

**Objective:** Patients with well-developed coronary collateral circulation (CC) usually have low mortality, improved cardiac function, and reduced infarct size. Currently, there is conflicting evidence on the association between traditional cardiovascular risk factors (diabetes, hypertension, and smoking habit) and CC.

**Design:** We performed a meta-analysis of case-control studies to better understand such associations.

**Data Sources:** We searched the MEDINE, EMBASE, and Science Citation Index databases to identify relevant studies.

**Eligibility Criteria for Selecting Studies:** Case control studies reporting data on risk factors (smoking habit, hypertension, and diabetes mellites) in comparing cases between poor CC and well-developed CC groups. Well-developed CC was the primary outcome of this meta-analysis

**Data Extraction and Synthesis:** Relevant data were extracted by two independent investigators. We derived pooled odds ratios (ORs) with random effects models. We performed quality assessments, publication bias, and sensitivity analysis to ensure the reliability of our results.

**Results:** In total, 18 studies that had 4,746 enrolled patients were analyzed. Our results showed that hypertension and smoking habit did not (OR = 0.94, 95% CI: 0.75–1.17, *p* = 0.564 and OR = 1.00, 95% CI: 0.84–1.18, *p* = 0.970, respectively), and diabetes did (OR = 0.50, 95% CI: 0.38–0.67, *p* = 0.00001) affect the development of CC.

**Conclusion:** Unlike hypertension and smoking habit, diabetes was associated with poor CC formation.

**Trial Registration Number:** https://www.crd.york.ac.uk/PROSPERO/display_record.php?RecordID=87821, identifier: CRD42018087821.

## Strengths and Limitations of This Study

-This is the largest meta-analysis to study the relationship between traditional risk factors and coronary collateral circulation.
-In order to give a solid conclusion, sensitivity and subgroup analyses were performed.
-Along with real operators/equipment, the definition/identification of the presence or absence of coronary collaterals can vary between different studies.

## Background

Traditional cardiovascular risk factors, including diabetes mellitus, smoking habit, and hypertension are associated with increased cardiovascular morbidity and mortality. These risk factors are predictors of poor clinical outcomes in patients with myocardial infarction, stroke, and peripheral artery diseases (1–3). Collateral circulation (CC) is beneficial in reducing cardiac ischemia and infarction in cases of chronic stenosis or acute occlusion. Well-developed coronary CC is associated with improved cardiac function and decreased instances of cardiac mortality (4, 5). Coronary collaterals are arterio-arterial anastomoses that develop neonatally and are present at birth (6). These anastomoses can remodel into arteries up to 20-fold larger in diameter than the original arterioles when a coronary artery is occluded. However, many acquired factors affect its arterial remodeling, which ultimately manifests as differences in the quality of collateral circulation in patients. Despite these beneficial effects, a large proportion of patients lack well-developed CC. Furthermore, an effective treatment to promote coronary CC has thus far been limited to humans (7). A number of studies have demonstrated that traditional cardiovascular risk factors can impair the remodeling of CC. Moore et al. found that traditional cardiovascular risk factors were associated with the premature rarefaction of the coronary CC, leading to greater cardiac ischemia (8). However, in comparison to the findings of Moore et al., some researchers have reported nearly opposite results (9, 10). The number of collateral circulation is also related to the degree of coronary artery obstruction. Given the conflicting evidence of the association between traditional cardiovascular risk factors (e.g., smoking habit, hypertension, and diabetes mellitus) and coronary collaterals, we performed a meta-analysis of case-control studies to better understand such relationships in patients with coronary artery chronic total occlusion. This is the largest meta-analysis to study the relationship between traditional risk factors and CC.

## Method

This meta-analysis is presented in accordance with the Preferred Reporting Items for System Reviews and Meta-Analyses (PRISMA) Statement and has been registered with the International Prospective Register of Systematic Reviews (CRD42018087821) (11). The protocol of this meta-analysis has been described previously (12).

### Search Strategies

We searched the MEDLINE, EMBASE, and Web of Science databases to identify relevant studies published from inception of those databases to January 1, 2021. We used the “coronary collaterals” as the key words, “collateral circulation” as the MeSH terms to identify relevant studies.

### Studies

Studies were included in our analyses if (1) they reported the extent of coronary CC, describing it as well-developed or poorly developed; (2) they reported data on risk factors (smoking habit, hypertension, and diabetes mellites) in comparing cases between poor CC and well-developed CC groups; (3) they were case-control studies; and (4) they contained sufficient information (for instance, the sample size of each group, the number of smokers in different groups, etc.). Studies were excluded if (1) they reported only the *p*-values of the data of interest without any numerical values in each group; (2) they were not published in English peer-reviewed journals; and/or (3) they presented data that were previously published.

### Exposure Factors and Grades of CC

Traditional coronary risk factors (smoking habit, diabetes mellitus, and hypertension) were defined as exposure factors. We used the Rentrop scoring system to grade CC (13). A Rentrop score of 2 or 3 was defined as well-developed CC, while a Rentrop score of 0 or 1 was classified as poor CC.

### Data Selection

Data selection was conducted independently (JY Pei and XP Wang).

Disagreements were resolved through discussion with all authors or by consulting a third author (ZH Xing). We selected the following data from the included articles: study characteristics (e.g., first author, publication date), the characteristics of included participants (e.g., age, sex, smoking habit, diabetes, and hypertension), the definition of coronary CC, and the risk factors of coronary CC. We used the Review manager and Stata to organize included articles and data.

If quantitative analysis was not appropriate, we performed a narrative, qualitative summary. Results are presented both in the text and in tables.

### Newcastle-Ottawa Scale

We assessed the methodological quality of the included studies by the Newcastle-Ottawa Scale. A maximum of two stars could be given for comparability measurements. A star system of the scale (range, 0–9) was used in the current study.

### Statistical Analysis

The relationship between cardiovascular risk factors (hypertension, diabetes, and smoking habit) and CC in patients with chronic coronary occlusion was expressed in terms of the odds ratio (OR) and its 95% confidence interval (CI). An OR >1 indicated that the studied factors were positively correlated with the formation of CC, while an OR <1 indicated that the studied factors were negatively correlated with the formation of CC. *P*-values < 0.05 were considered statistically significant. Heterogeneity was assessed by the Cochran *Q*-test, and statistical heterogeneity was summarized by the *I*^2^, which quantifies the percent of variation in study results that is because of heterogeneity rather than to chance. When *I*^2^ was <25%, the heterogeneity was small, and a fixed-effect model will be used. When *I*^2^ ≥ 25%, the included studies were considered to have moderate heterogeneity, the *I*^2^ ≥ 50%, there is a significant heterogeneity (14). When the moderate or significant heterogeneity was found, the random-effects model will be used. Accordingly, a sensitivity analysis was also conducted to verify whether the effect was stable. Random-effects and fixed-effect models were used to calculate the respective effect, and whether the observed result was affected by the statistical method; each study was excluded, and the effect of the remaining studies was recalculated to determine whether the observed results were affected by any individual study. The Begg and Egger methods were used to verify whether there was publication bias.

## Results

### Included Studies

**Figure 1:**
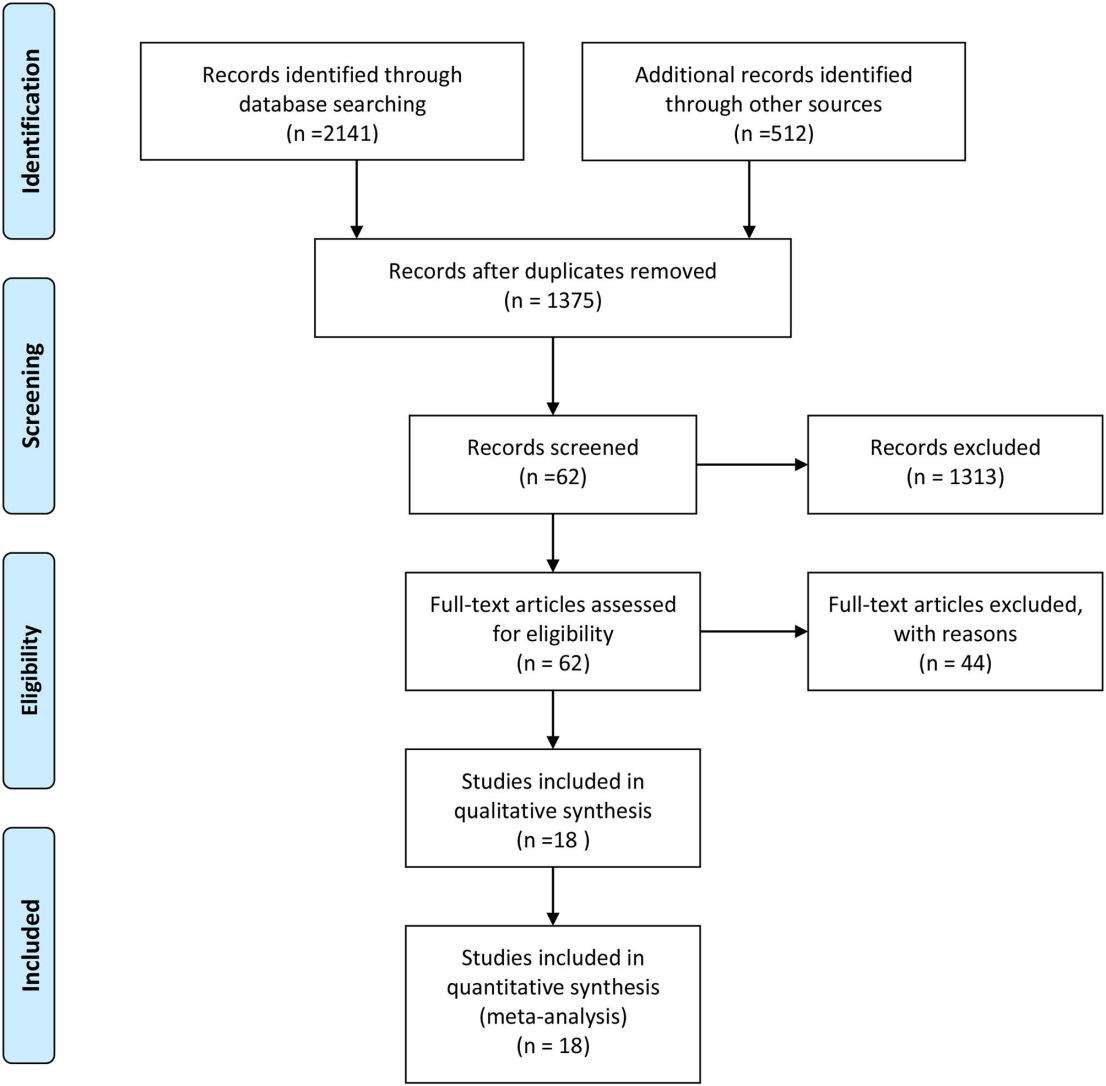
Literature screening flowchart.

An outline of the screening process is shown in Figure 1. A total of 2,653 documents were identified manually by searching the MEDLINE, Embase, and Web of Science databases, as well as other sources. After the removal of duplicates, 1,375 articles were retained. Subsequently, after screening the titles and abstracts of the literature, 1,313 studies were excluded. Thus, a total of 62 relevant articles were identified. Finally, after reading the full text, 44 articles were excluded [3 non-English articles, 33 non-coronary chronic total occlusions (CTO), 3 similar articles by the same author, and 5 unrelated research], and 18 articles were included for further analyses.

A total of 4,746 CTO patients were enrolled in the selected studies, including 2,928 (61.7%) patients with hypertension, 1907 (40.2%) patients with diabetes, and 2,146 (45.2%) patients with a history of smoking. Most of these studies were cross-sectional studies that study the relationship between blood biochemical indicators and coronary collateral circulation. These biochemical indicators have been shown to be related to the severity of cardiovascular disease in previous studies. In most studies, male patients accounted for the majority. This might be because the formation of coronary collateral circulation was related to the severity of coronary atherosclerosis, and male patients usually have more severe symptoms than female patients (Table 1). The baseline patient's characteristics was shown in Table 2.

**Table 1:** General information of research literature.

**Research name**	***N***	**Mean age (G/P, years)**	**Male (G/P, %)**	**Research purposes**
Açar et al. (15)	294	59/61	84/82	Relationship between platelet and lymphocyte ratio and CC
Balli et al. (16)	430	64/62	61.5/63	Relationship between thyroid function and CC
Baykan et al. (17)	163	61/59	72/81	Relationship between arterial elasticity and CC
Ceyhan et al. (18)	113	58/61	72/70	Relationship between ACE gene polymorphism and CC
Perera et al. (19)	58	59/60	73/80	CC and posterior restenosis
Duyuler et al. (20)	88	65/63	80/76	Relationship between pleiotrophin and CC
Erdoan et al. (21)	179	62/64	85/78	Relationship between bilirubin and CC
Erdoan et al. (22)	148	65/61	82/8.	Relationship between QRS fragmentation wave and CC
Kadi et al. (23)	299	65/62	72/78.6	Relationship between glomerular filtration rate and CC
Kalkan et al. (24)	274	61/59	80/85	Relationship between neutrophil-lymphocyte ratio and CC
Nacar et al. (25)	138	60/60	53/62	Relationship between neutrophil-lymphocyte ratio and CC
Sarli et al. (26)	203	68/68	44/48	Relationship between gamma glutamyl transferase and CC
Shen et al. (27)	718	66/64	70/78	Relationship between hypertension and CC
Shen et al. (28)	434	67/64	62/60	Relationship between glycated hemoglobin and CC
Sögüt et al. (29)	189	61/63	81/77	Relationship between vitamins A/E and CC
Sun et al. (30)	478	66/64	71/79	Relationship between C-reactive protein, renal function, diabetes, and CC
Yang et al. (31)	324	68/56	61/85	The relationship between CXCR4 and CC
Yang et al. (32)	216	61/62	87/81	CC and posterior restenosis

*G, good collateral circulation; P, poor collateral circulation*.

**Table 2:** Baseline patients characteristics.

**Research name**	**Well-developed CCC (N)**	**Poorly developed CCC(N)**	**Body mass index, kg/m^**2**^**	**Hyperlipidemia, (%)**	**Previous myocardial infarction, (%)**	**LVEF, %**	**SYNTAX score**	**Position of chronic total occlusion lesion**			**Rentrop collateral grades**				**Number of diseased coronary vessels**
								**LAD, (%)**	**LCX, (%)**	**RCA, (%)**	**0, (%)**	**1, (%)**	**2, (%)**	**3, (%)**	
Açar et al. (15)	131	163	27.8	/	/	55%	/	41.80%	20.10%	38.10%	14%	41.50%	27.90%	16.70%	1.70
Balli et al. (16)	313	117	/	49.30%	/	46%	/	/	/	/	/	/	/	/	1.99
Baykan et al. (17)	85	78	27.4	36.81%	/	/	22.2	36.19%	11.66%	52.15%	/	/	/	/	/
Ceyhan et al. (18)	67	46	26.8	61.06%	51.32%	/	/	39.82%	24.78%	45.13%					2.07
Perera et al. (19)	25	33	/	/	29.30%	65%	/	51.70%	20.70%	27.60%	/	/	/	/	/
Duyuler et al. (20)	58	30	/	/	/	50%	/	26.10%	46%	28.40%		35.23%	30.68%	34.09%	/
Erdoan et al. (21)	110	69	28.6	74%	16.20%	47%	/	45.25%	11.18%	40.78%	/	/	/	/	1.2
Erdoan et al. (22)	93	55	/	75%	/	49%	/	49.32%	9.46%	42.57%	/	/	/	/	1.2
Kadi et al. (23)	206	93	/	39.46%	/	/	/	43.81%	23.75%	49.83%	/	/	/	/	2.16
Kalkan et al. (24)	118	156	27.8	/	/	55%	17.46	41.60%	17.52%	40.88%	/	/	/	/	1.7
Nacar et al. (25)	72	66	/	/	/	45%	/	/	/	/	/	/	/	/	/
Sarli et al. (26)	104	99	28	/	/	/	/	/	/	/	11.82%	37.43%	44.33%	6.40%	/
Shen et al. (27)	440	278	25.1	30.78%	/	/	/	/	/	/	/	/	/	/	2.06
Shen et al. (28)	294	140	25.4	59.88%	/	/	/	/	/	/	/	/	/	/	2.15
Sögüt et al. (29)	93	96	26.1	42.32%	/	/	/	55.56%	22.75%	47.09%	/	/	/	/	1.85
Sun et al. (30)	292	186	/	45.81	/	/	/	/	/	/	/	/	/	/	2.16
Yang et al. (31)	78	78	25.6	47.43%	/	49%	/	/	/	/	/	/	/	/	/
Yang et al. (32)	132	84	25.9	24%	/	50%	/	35%	18%	47%	/	/	/	/	2.4

### Newcastle-Ottawa Scale

The Newcastle-Ottawa Scale was used to evaluate the quality of the included studies. Each of the included studies had a score above seven points, and thus, all these studies were included in our analyses.

### Outcomes

#### Hypertension and CC

**Figure 2:**
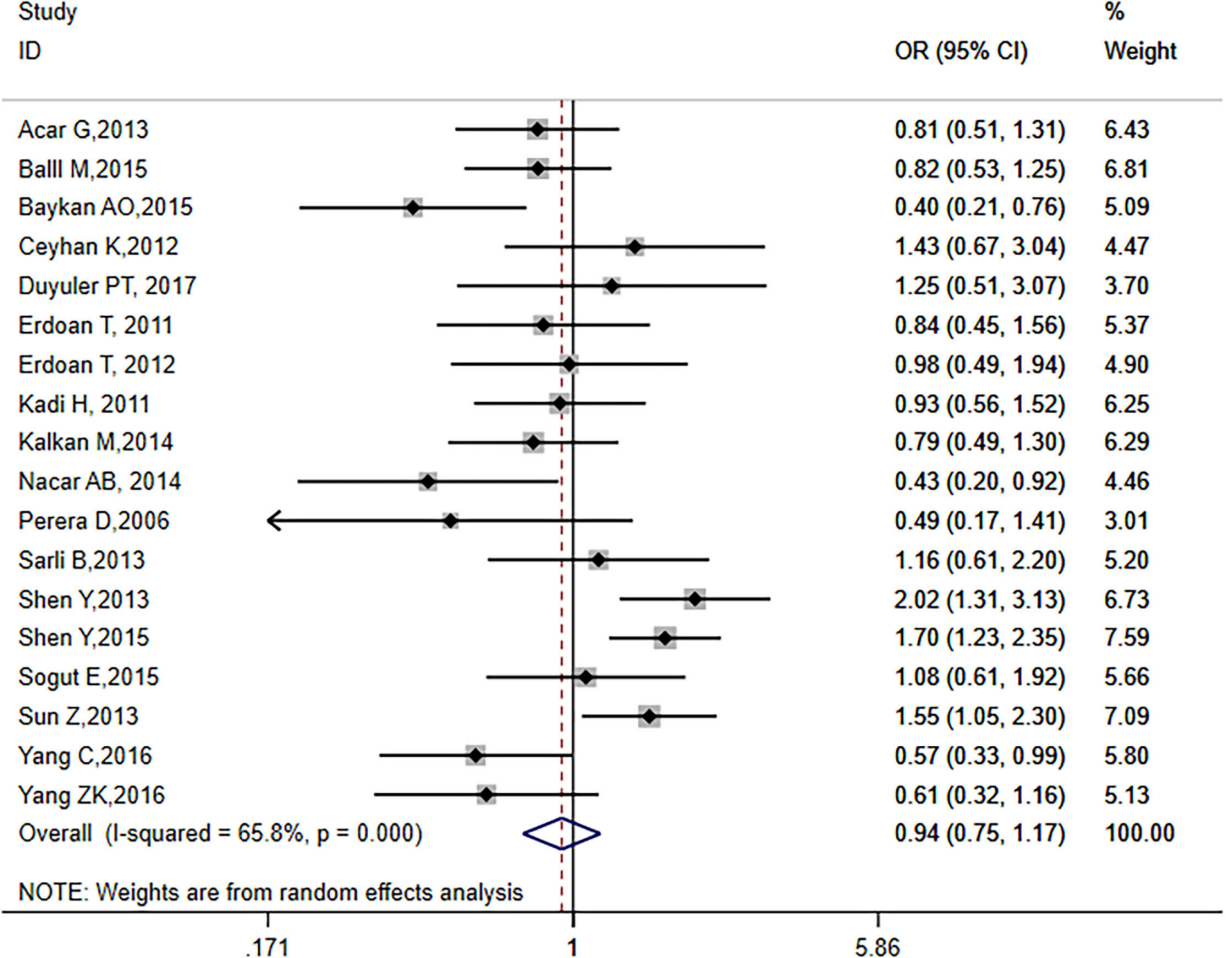
Relationship between hypertension and CC in patients with CTO.

The analysis included 18 articles, representing a total of 4,746 patients, including 2,928 hypertensive patients. The *I*^2^ score was 65.8% (*p* = 0.0001), indicating that the included literature was heterogeneous, thus requiring a random-effects model (OR = 0.94, 95% CI: 0.75–1.17, *p* = 0.564; Figure 2). The effect value was close to 1, suggesting that hypertension had no effect on the formation of CC in CTO patients.

#### Diabetes and CC

**Figure 3:**
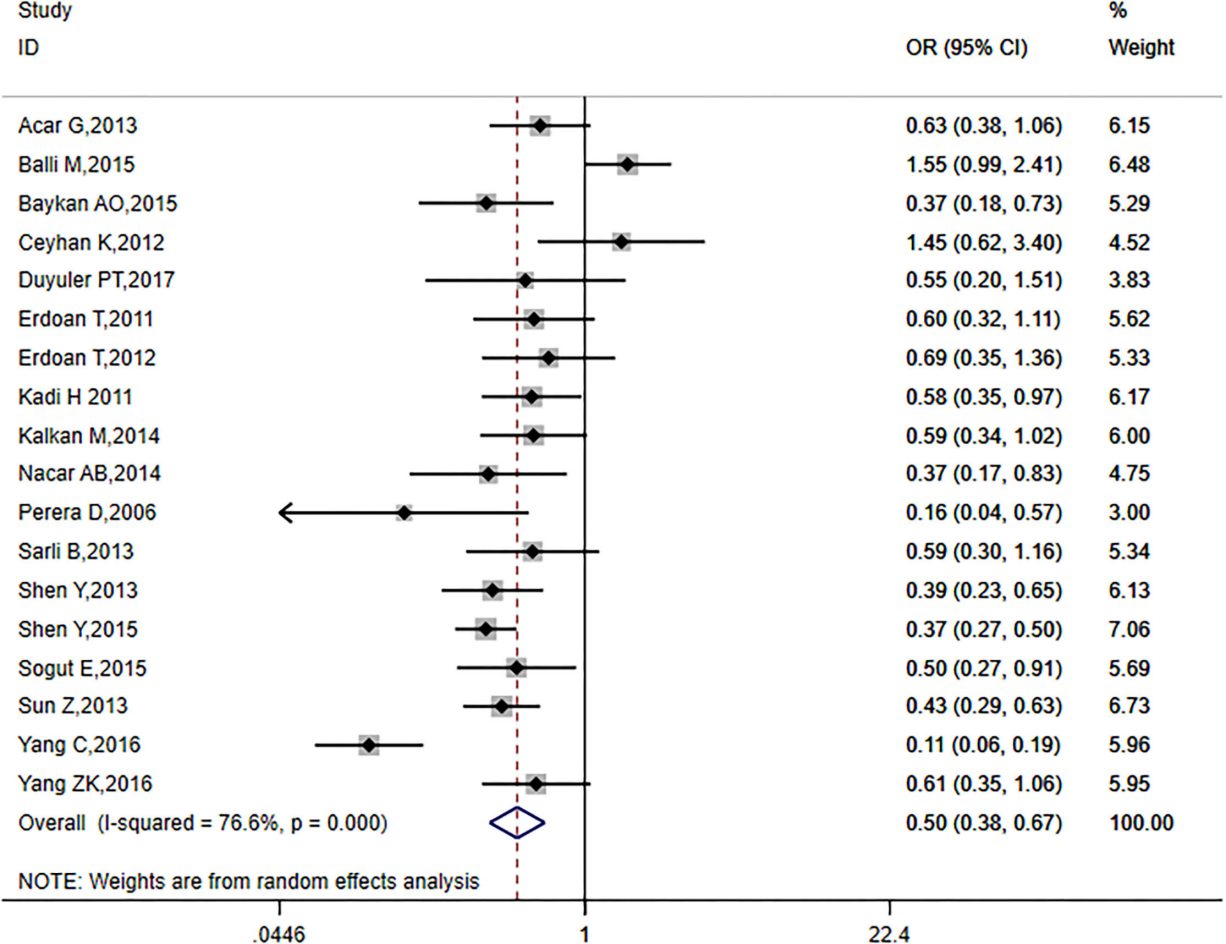
Relationship between diabetes and CC in patients with CTO.

The analysis included 18 articles with a total of 4,746 patients, including 1907 (40.2%) diabetic patients. *I*^2^ = 76.6% (*p* = 0.0001) indicated that the included literature was heterogeneous; thus, requiring a random-effects model (OR = 0.50, 95% CI: 0.38–0.67, *p* = 0.00001; Figure 3). These results suggest that CC formation was inhibited in patients with diabetes mellitus.

#### Smoking Habit and CC

**Figure 4:**
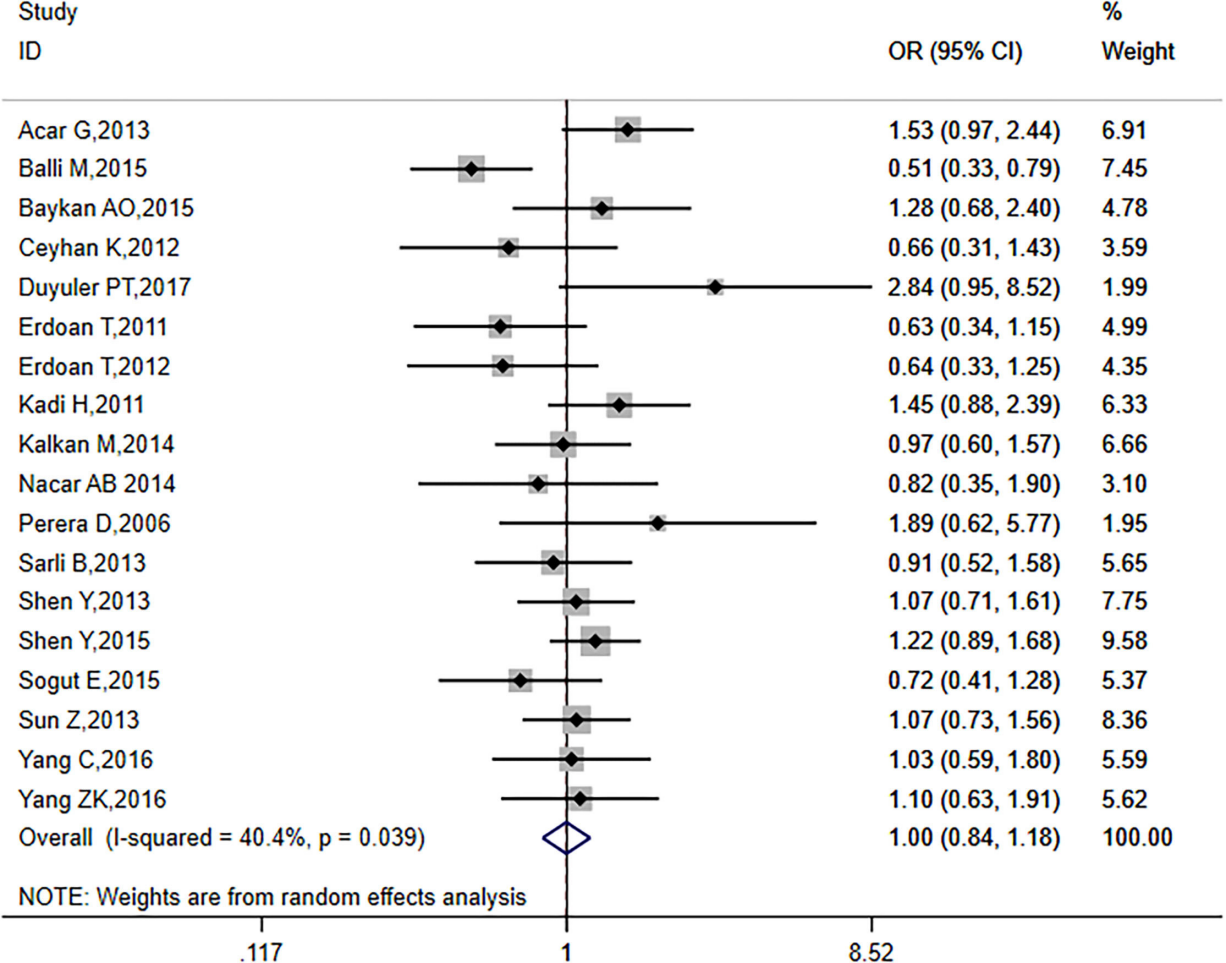
Relationship between smoking and CC in patients with CTO.

The analysis included 18 articles with a total of 4,746 patients, including 2,146 (45.2%) patients with a history of smoking. *I*^2^ = 40.4% (*p* = 0.06) indicated that the included literature showed significant heterogeneity. Therefore, a random-effects model was used (OR = 1.00, 95% CI: 0.84–1.18, *p* = 0.970; Figure 4). These results suggest that smoking habit did not affect the formation of CC in CTO patients.

#### Sensitivity Analysis and Publishing Bias

The use of a fixed-effect model showed that the statistical methods did not influence the observed results (Hypertension and CC: OR = 1.03, 95% CI: 0.91–1.16, Supplementary Figure 1), (Diabetes and CC: OR = 0.49, 95% CI: 0.43–0.56, *p* = 0.0001, Supplementary Figure 2), (Smoking habit and CC: OR = 1.01, 95% CI: 0.89–1.14, Supplementary Figure 3). Subsequently, each study was excluded, and the effect of the remaining studies was recalculated to determine whether the observed results were affected by any individual study. No study was found to alter the effect size.

The Begg and Egger methods were used to test publication bias. No publication bias was observed for hypertension (*p* = 0.225, Begg method; *p* = 0.142, Egger method), diabetes (*p* = 0.695, Begg method; *p* = 0.663, Egger method), and smoking habit (*p* = 0.596, Begg method; *p* = 0.996, Egger method).

## Discussion

This is the largest meta-analysis to study the relationship between traditional risk factors and CC, which included 18 case-control studies that had 4,746 CTO patients. No association was found between hypertension or smoking habit and CC in patients with CTO. However, CC was found to be inhibited in diabetic patients.

In the 1970s, the measurement of functional collaterals of coronary arteries in *vivo* was performed for the first time, showing the “direct relationship between the appearance and functional performance of coronary collaterals in bypass surgery (33).” Rentrop et al. proposed a method of intracoronary angiography to divide the coronary collateral circulation into four levels, which we used in this research (13). But this method still has certain limitations. This method was a qualitative judgment method, and it was not suitable for epicardial collaterals. In recent years, a quantitative assessment method of coronary collateral function based on the measurement of coronary occlusion pressure has emerge, which called the collateral flow index (CFI) (34, 35). This method can be used as a reference method for evaluating functional collaterals in patients with chronic stable coronary heart disease (36, 37). It has been confirmed that CFI >0.20–0.25 was related to the absence of signs of ischemia on the intra-coronary electrocardiogram during 1 min of coronary balloon occlusion (38, 39).

One characteristic of hypertension is an increase in the stiffness of blood vessels. This stiffness may increase the sheer force of the bloodstream to the collaterals. The increase in shear force is likely to increase the activity of endothelial nitric oxide synthase (eNOS), leading to an increase in nitric oxide (NO) synthesis (40). However, hypertension is associated with vascular dysfunction (i.e., decreased vascular compliance). In patients with hypertension, angiotensin II can inhibit angiogenesis, but can also enhance NAD (P) H oxidase activity, leading to increased vascular wall reactive oxygen species and superoxide. This then leads to the uncoupling of eNOS, which inhibits the synthesis of NO, resulting in vascular endothelial dysfunction. At the cellular level, the relationship between hypertension and CC may be more complicated, both promoting and inhibiting the formation of CC. Thus, the relationship between hypertension and CC can be complicated—promoting and inhibiting the formation of CC (41). There are a few observational clinical studies on the association between hypertension and CC, but these studies have produced varied conclusions (40, 42).

Diabetes is a relatively important risk factor for coronary heart disease, and it can lead to poor short-term and long-term cardiovascular outcomes (43). Abaci et al. studied 205 diabetic patients with severe coronary artery stenosis and found that coronary artery CC was poorly formed in these patients than in non-diabetic patients with severe coronary artery stenosis (44). Subsequent studies have also found that patients with diabetes exhibited poor CC formation (45). However, in sharp contrast to the above studies, several studies have drawn completely opposite conclusions (46). Similar to the findings of studies that examined patients with hypertension, there was a considerable heterogeneity among patients enrolled in these studies. Diabetes may lead to severe coronary lesions, which may aggravate tissue ischemia and promote CC formation. In the current study, only patients who were diagnosed with CTO were included, thus avoiding differences in the results of the relevant studies due to differences in the degree of vascular stenosis. CC in patients with diabetes and CTO can be related to various clinical, biochemical, and angiographic factors. It involves multiple mechanisms, such as an increase in the serum levels of glycated albumin, cystatin C, and adipokine C1q tumor necrosis factor related protein 1 (47).

Revascularization of patients with chronic complete coronary occlusion can significantly improve the patient's left ventricular function and reduce the risk of cardiovascular events (48) and current evidence favors coronary artery bypass grafting as the preferred revascularization modality for type 2 diabetic patients with multivessel coronary disease, which is likely to reflect the more complete revascularization and global protection provided by arterial conduits against rapid atherosclerosis progression in PCI and untreated segment (49, 50). Jane et al. found that after revascularization of CTO patients, patients with better collateral circulation have a lower risk of cardiovascular events (51). Recent studies have found that PCI can reduce the risk of cardiovascular events and improve the survival rate of non-diabetic CTO patients, but it has no obvious effect on CTO patients with diabetes (52). However, some clinical studies have found that vascular recanalization can indeed improve the survival rate of CTO patients with diabetes (53). Therefore, whether revascularization should be performed on CTO patients should not only rely on clinical symptoms, but also comprehensively consider the patient's vascular conditions, of which the quality of collateral circulation is a very important factor.

Moreover, tobacco consumption may both promote and inhibit the formation of CC. Heeschen et al. found that nicotine promoted the adhesion of monocytes to endothelial cells and thus, may promote the development of CC (54). However, a long history of smoking can lead to endothelial dysfunction and impaired monocyte migration. Such impaired endothelial function and the dysfunction of inflammatory cells caused by cigarette consumption can inhibit the positive remodeling of CC to some extent.

### Limitation

The current study has a few limitations. First, this is a meta-analysis of observational studies, and thus, it was not possible to consider all confounding factors between groups with and without good CC formation, which may affect the evaluation of our results. There are many factors that can affect the formation of coronary collateral circulation, such as age, weight, ethnicity, body mass index or cholesterol level. The data obtained from the original study can only show the average age or average weight of all patients. As far as we know, we cannot adjust in statistical methods based on these data, so we did not adjust age and weight in the statistical process. As for race, because most of the included studies are single-center case-control studies, the original article did not provide exact race data, these may have some influence on the results. Additionally, along with real operators/equipment, the definition/identification of the presence or absence of coronary collaterals can vary between different studies, and therefore, such differences can affect the quality assessment of CC. And we used the Rentrop scores to evaluate the quality of CC, not the CFI, because Rentrop scores was used in most studies. We were unable to obtain relevant data from these original studies, so we did not perform the inter and intra-observer variability analyses. The OR value of hypertension and smoking habit was 0.92 and 1.01, respectively. The effect values and 95% CI of these two variables were very close to 1. However, the effect values and 95% CI for diabetes was close to 0.5. Even if there were confounding factors that may have affected the outcome measures, they are unlikely to overturn the observed results of this study since our meta-analysis incorporated a large population base, and subgroup/sensitivity analyses were used to verify the reliability of our conclusions. Since the included studies were all case-control studies, data of patient's hypertension stage cannot be collected from the original article, so we cannot perform the analysis of the impact of hypertension stage and collateralization. As well for diabetes, data of patient's hypertension stage cannot be collected from the original article, we cannot perform the analysis of the HbA1c and collateralization status in diabetics either. Second, despite a detailed search of different databases, some studies could have been missed during the initial search. Finally, the bulk of the studies included in our meta-analysis was performed in China and Turkey, which may limit the generalizability of our results in other (European, American, and African) populations.

## Conclusion

This meta-analysis found that hypertension and smoking habit were not associated with CC formation. However, diabetes was associated with poor CC formation.

## Data Availability Statement

The original contributions presented in the study are included in the article/Supplementary Material, further inquiries can be directed to the corresponding author/s.

## Author Contributions

ZX designed the study and provided methodological expertise. JP and XW data selection was conducted independently and relevant studies were searched by 2 independent investigators. JP and ZX drafted the manuscript. All authors have read, provided critical feedback on, and approved the final manuscript.

## Funding

This work was supported in part by National Science Foundation of China Project No. 82000298 and Natural Science Foundation of Hunan Province 2021JJ40883 to ZX.

## Conflict of Interest

The authors declare that the research was conducted in the absence of any commercial or financial relationships that could be construed as a potential conflict of interest.

## Publisher's Note

All claims expressed in this article are solely those of the authors and do not necessarily represent those of their affiliated organizations, or those of the publisher, the editors and the reviewers. Any product that may be evaluated in this article, or claim that may be made by its manufacturer, is not guaranteed or endorsed by the publisher.
