# Supplementary material for: Traditional Cardiovascular Risk Factors and Coronary Collateral Circulation: A Meta-Analysis
Source: Front Cardiovasc Med. 2021 Nov 3;8:743234. doi: 10.3389/fcvm.2021.743234 (PMC8595282; doi:10.3389/fcvm.2021.743234)
Supplement: Supplementary file 1 [file Data_Sheet_1.docx]

**Traditional cardiovascular risk factors and coronary collateral circulation: a meta-analysis**

**Junyu Pei MD^1^, Xiaopu Wang MD^1^, Zhenhua Xing MD^2^***

**1 Department of Cardiovascular Medicine, The Second Xiangya Hospital, Central South University, Changsha, Hunan 410011, China**

**2 Department of Emergency Medicine, Second Xiangya Hospital, Central South University, Changsha, 410011, China.**

***Corresponding author:**

**Zhenhua Xing M.D (xing2012x@csu.edu.cn).**

**Department of Emergency Medicine,**

**Second Xiangya Hospital, Central South University,**

**Changsha, 410011, China**

**Phone number: +861584714930**

**Supplementary Information**


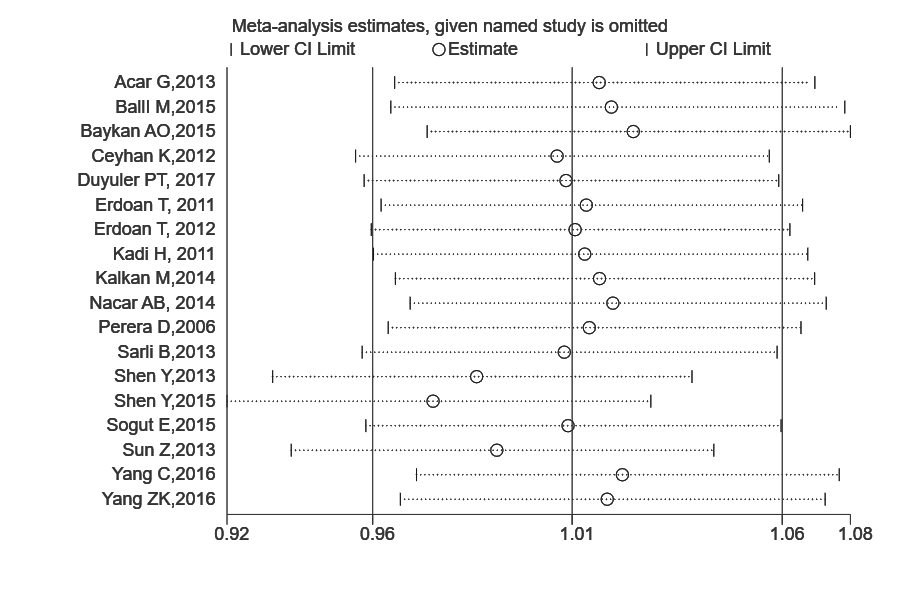
Figure 1. Evaluation of whether a single study altered outcomes. Points and vertical lines correspond to individual studies, representing the OR and 95% CI of the remaining studies after deletion of the study.


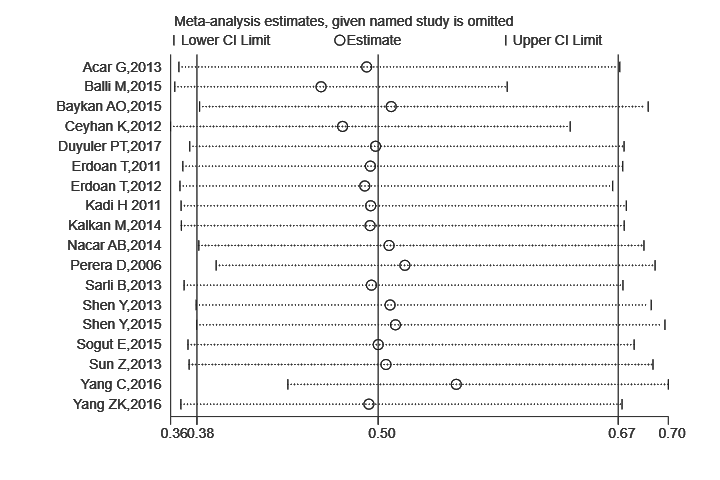
Figure 2. Evaluation of whether a single study altered outcomes. Points and vertical lines correspond to individual studies, representing the OR and 95% CI of the remaining studies after deletion of the study.


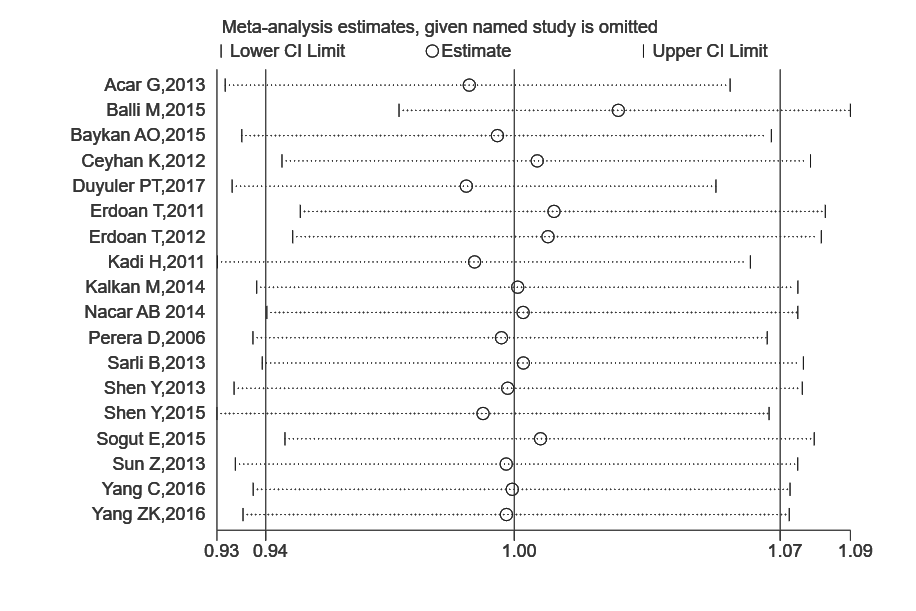


Figure 3. Evaluation of whether a single study altered outcomes. Points and vertical lines correspond to individual studies, representing the OR and 95% CI of the remaining studies after deletion of the study
